# Stress, Burnout, and Low Self-Efficacy of Nursing Professionals: A Qualitative Inquiry

**DOI:** 10.3390/healthcare8040424

**Published:** 2020-10-23

**Authors:** Luis Miguel Dos Santos

**Affiliations:** Woosong Language Institute, Woosong University, Daejeon 34514, Korea; luisdossantos@woosong.org; Tel.: +82-010-3066-7818

**Keywords:** bullying, burnout, discrimination, registered nurse, self-efficacy, stress

## Abstract

Nursing professionals face a high level of stress and burnout due to overloaded responsibilities, which may cause a low level of self-efficacy. From the perspective of nursing professionals, the research aims to understand what are the sources of stress and burnout which would reduce the self-efficacy and the unbalanced patient ratio and how would nursing professionals describe their experiences, sources of stress and burnout, and self-efficacy. Based on the snowball sampling strategy, 60 nursing professionals were invited for qualitative research data collection. Based on the lens of the self-efficacy approach, the results indicated that the environmental factors, including workplace bullying, family stress, misunderstanding of public members, and personal development and career enhancement took important roles in increasing their stress and burnout and in reducing their self-efficacy. The outcomes of this study discovered the social status and discrimination toward nursing professionals. Government leaders, policymakers, and researchers should take this research as an opportunity to reform their policy for human resource management and education for the respectfulness of medical and nursing professionals in the public health system.

## 1. Introduction

### 1.1. Purpose and Background of the Study

Nursing professionals make up one of the most important groups in the public health system. However, previous studies indicated that stress, burnout, being overloaded with responsibilities, social bias, and stigma [1,2] may negatively influence their professional position and status in society. In this context, researcher advocated that the social environment, cultural background, and personal characteristics might impact the results and experiences regarding stress and burnout. Researchers in the field of psychology strive to understand the relationship between stress, burnout, and occupational experiences that occurs in their social and cultural environment [3,4]. Also, how would the causes of stress and burnout originating from their social and cultural environment influence their self-efficacy [5], in this case, as nursing professionals?

Currently, among nurses in South Korea, regardless of their level, specialization, gender, and registration, workplace stress and burnout have been associated with health issues such as negative feelings, depression, and stress. Nursing and medical staff always experience stress due to being overloaded with duties, responsibilities, working hours, and conflicts between different parties, such as patients, coworkers, members of the public, governments, and the media. Besides the problems in the workplace, it is not uncommon for there to also be stress and problems within the family environment. Family members might dislike the working responsibilities and environment of nurses and medical staff. Therefore, stress and burnout from different directions may increase mental distress and, as a consequence, result in greater staff turnover and attrition.

According to Bandura, self-efficacy [6,7] may have an important role not only in how individuals think about themselves but also on whether they successfully pursue their interests, goals, and achievements within the plans they made for the future and their career development. The public health and nursing profession is one of the occupations hardest hit by stress, burnout, overload of responsibilities, social bias, and stigma from members of the public, so it is important to understand how these individuals perceive their situation and how they react to different situations in society.

The purpose of this study was to understand and explore whether nursing professionals’ self-efficacy may be influenced by social and cultural (i.e., environmental) factors and what are the main sources of these factors among nursing professionals in South Korea. Although the connections between self-efficacy, social and cultural elements, personalities, and external influences seem to be predictable and untestable without additional research activities, understanding the connections between self-efficacy and additional reasons and factors of stress and burnout may expand the knowledge of the position of self-efficacy for nursing professionals. In short, it is important to understand the self-efficacy, stress, burnout, and well-being problems faced by nursing professionals [8,9]. For decades, the South Korean public health system faces a significant shortage of nursing professionals. Due to the unbalanced medical and nursing professional-to-patient ratio, nursing professionals face excess levels of stress and burnout due to being overloaded with responsibilities brought about by this unique situation [10]. Therefore, the researcher tried to understand, through the lens of the self-efficacy approach, how nursing professionals describe their experiences, particularly the sources and reasons for their stress and burnout [11,12,13,14,15].

The current study was guided by two research questions:

(1) How does the career pathway of the nursing profession impact the stress, burnout, social bias, and stigma of women nursing professionals in South Korea?
(2) What are the sources of stress and burnout that reduce the self-efficacy of women nursing professionals in South Korea?

### 1.2. Brief Literature Review

Currently, there are about 338,600 registered nurses in South Korea. However, due to the high levels of stress and negative working conditions, less than 210,000 nursing practitioners are currently working in the health and welfare sectors and facilities [16]. In other words, more than 100,000 registered nurses are not working in the medical and nursing profession [16] due to various reasons, such as poor working environment, overload of responsibilities, and traditional family structure [17]. In order to encourage these registered nurses to return to the medical and nursing profession to tackle the ageing population, increasing demands of medical professional–patient ratio, the related Korean ministry established policies which attracted nearly 900 registered nurses. However, this was not enough. Although foreign nursing professionals may practice nursing in South Korea, most need to conduct degree-equivalent reports (i.e., degree evaluation report) and to pass regional-level exams in order to obtain practicing registration. As a result, only 36 nursing professionals registered under this scheme [16]. A recent report [18] indicated that, in South Korea, there were at least 237,744 registered nurses, 101,450 doctors, 25,315 dentists, 19,959 oriental medicine practitioners, and 417,800 pharmacists in the healthcare system in 2017. As of the year 2000, there were more than 50 education institutions offering a Bachelor of Science in Nursing (BSN) degree for prospective registered nurses. Besides the traditional BSN programs run in secondary schools, the BSN degree can be achieved from the University of the Air after completion of a three-year-long associated degree resulting in attainment of a Registered Nurse status (RN-BSN). Although the South Korean government established schemes for in-service nurses’ professional development and made available additional funding and resources for nursing schools and programs, the policies did not solve the high level of medical and nursing professional-to-patient ratio [19].

According to previous studies [20,21], one of the characteristics of the South Korean managerial style is its family orientation and power centralization. In other words, the upper levels of leadership always control decision-making powers and lower-level team members need to follow policies without having been consulted [22]. Another significant characteristic of the South Korean leadership style is personalization. In other words, a small group of board members control human resources management, the decision-making process, financial decisions, and governmental relations, with lower-level employees having no right to comment [21,23,24]. More importantly, women usually do not have the right to access upper management leaders due to the prevalent gender bias. Although the nursing profession mainly comprises women, due to the assistance orientation of their positions and the gender issues they face, nurses usually do not have the right to comment on any decisions made by upper management teams. The presence of women in leadership teams is uncommon in South Korea, even in the field of nursing practices. Due to gender bias, many hospitals appoint male managers from other departments and divisions to manage nursing teams [25,26].

Traditionally, nursing has been a women-oriented profession in South Korea [27,28,29]. After the first westernized nursing school was established during the mid-1900s, nursing education has been established under a westernized curriculum and instruction. However, the public’s perception of the nursing profession was not very positive. Most believed nurses were assistants and individuals who failed to enter medical school. More importantly, due to social bias against the nursing profession, it was not until mid-2015 that the government changed the college-level (i.e., three-year) nursing education curriculum to university level (i.e., four years). Therefore, discrimination and social bias against nurses were still significant [30,31].

A previous study [32] indicated that doctors, therapists, and even senior nursing professionals in South Korea always look down on nurses due to their low social status. From the traditional South Korean perspective, nurses are considered assistants and helpers of doctors, therapists, and patients. More than one-third of nursing students believed nurses should not have the right to access leadership positions but should remain lower-level team members [33].

A previous study [32] further indicated that nurses usually face challenging work schedules after marriage or childbirth. As a result, about 60 per cent of nurses reported muscle pains, skin troubles, headaches, insomnia, indigestion, and depression. In addition, another study [34] indicated that the on-shift working schedule also severely affects mental health, emotional state, and sleeping patterns of nursing professionals who work in hospitals. Particularly, during the peak seasons or during a disaster, medical and nursing professionals face additional pressures, stress, and burnout due to the extreme medical and nursing professional-to-patient ratio [35,36].

From the perspective of self-efficacy, a previous study [37] was conducted to understand the relationship between nursing professionals and medical staff’s burnout and emotional distress in South Korea. The results showed that nursing professionals and medical staff usually face problems with emotional distress, low levels of self-efficacy, gender concerns, and the ratio between patients and professionals. The research suggested that government leaders and policymakers should develop programs and schemes to increase self-efficacy and to reduce mental distress of nursing professionals and medical staff in order to maintain effectiveness of public health operation.

The relationship between self-efficacy and social support could be one of the solutions for nursing professionals’ emotional labor, burnout, and turnover decision. A previous study [38] investigated the role of self-efficacy and social support programs in areas of emotional distress and self-efficacy of 389 nurses in three hospitals in South Korea. The results indicated that social support might be one of the connections of self-efficacy and emotional distress of nursing professionals. Leadership in hospitals should focus on the development of social support groups and programs in order to increase nursing professionals’ self-efficacy.

### 1.3. Theoretical Framework: Self-Efficacy Approach

Self-efficacy [7] was one of the famous psychological approaches which indicated how individuals describe their experiences, understanding, and the reaction of their internal feelings and external environments. The self-efficacy approach refers to the personal beliefs of individuals in their capabilities to arrange, handle, motivate, organize, and conduct the series of behaviors required to achieve desired outcomes and results [7,39]. Perceived self-efficacy [40] involves understanding of personal responsibilities and is defined as an individual’s personal beliefs in and understanding of their capabilities to handle and manage the motivation, cognitive resources, and the series of behaviors required to assert control over outcomes and results in their lives [41].

Self-efficacy [7,39] is an individual’s personal belief and understanding that they have the capacities to be successful in their life experiences, tasks, assignments, and outcomes. It is important to understand that individuals may have a high level of self-efficacy in a particular situation [42,43], in this case, the capability to care for patients and to manage of their daily operations at the clinical level; however, this might not be the case when, for example, it comes to office politics and responsibilities that they are burdened with that they deem incongruous with their job description. Behaviors and coping strategies may depend upon their self-efficacy levels and mental situation [6,44].

**Figure 1:**
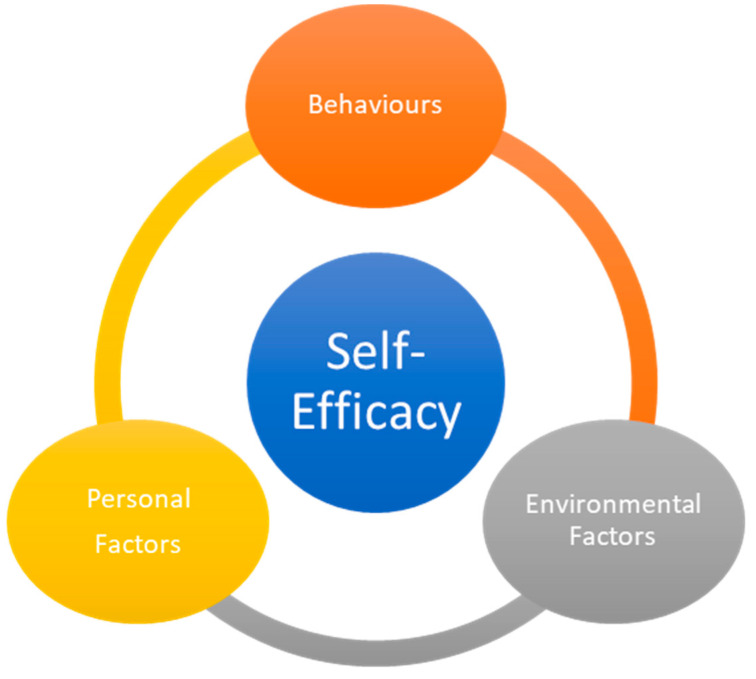
The triadic reciprocality.

Self-efficacy was based on Social Cognitive Theory [45,46,47,48,49,50] that defines and described how individuals might be influenced based on their thoughts, behaviors, feelings, motivations, and personal beliefs. For information on how individuals’ behaviors and actions are further described based on the model of causation, referred to as triadic reciprocality, please see Figure 1. Bandura’s triadic reciprocality [7,39,41,51] held that an individual’s capabilities and self-efficacy are interconnected to different elements and factors, which all interact with each other. These elements were (1) personal factors and elements, such as cognition, affect, and biological elements; (2) behaviors; and (3) environmental elements and factors.

## 2. Methodology and Its Application

The phenomenological approach and analysis [52] was used to gather, collect, and analyze the lived stories, experiences, self-efficacy, stress, and burnout among nursing professionals in South Korea. Without the qualitative interview sessions, the researcher could not collect the personal sharing and feedback from the participants.

### 2.1. Application of the Phenomenological Approach

Unlike the application of case study [53] or interpretative phenomenological analysis (IPA) [54], the phenomenological approach [52] tends to understand and explore a group of individuals’ common or shared experiences and lived stories of the social issues, problems, and phenomenon. In other words, a group of people in the same society, industry, profession, workplace, or community faced the same issue(s) but different experiences and lived stories. In order to expand the scope from a single or limited location, such as a hospital [53], the phenomenological approach tends to collect voices and data information from a group of individuals instead of a single location.

Moustakas [52] advocated that the phenomenological approach is applicable to understand individuals’ behaviors, sharing, lived stories, and life experiences. More importantly, based on the guideline of the phenomenological approach, in order to collect a wider perspective from different people and groups, researchers should collect data from at least 50 individuals and groups. In this study, 60 individuals were invited.

### 2.2. Participants and Recruitments

Sixty nursing professionals (i.e., with administrative, direct care to patient(s), and supervising experiences) at hospitals and clinics were invited. The participants in this study agreed to participate in this study. All of the participants had at least ten years of clinical experiences.

The study was conducted during Spring 2019. The snowball sampling tool [55,56] was employed for recruitment. First, based on the researcher’s personal network and connection, the researcher could invite five experienced nursing professionals in South Korea. The researcher first connected the potential participants with cellphone calls. After the participants orally agreed on the participation, the researcher emailed an invitation letter with the nature of the study, protocol, interview questions, content form and agreement, and the related materials to the participants. Third, both arranged a time for the social media-oriented interview session via computer social media chat (i.e., Kakao Chat (Kakao, Seoul, South Korea)). Fourth, after the participants completed the interview session(s), the researcher asked each to refer at least one nursing professional who a similar background (i.e., individuals who meet the requirements of this study) for expansion of this study. As a result, 60 nursing professionals were willing to join this study.

Some may argue that the number of the participants is not enough to reflect the overall performance of this study. First, many potential interviewees were not willing to share their experiences due to personal and occupational reasons. As mentioned above, based on the snowball sampling tool, participants should refer to at least one potential participant for the study. Based on the recruitment procedure, two potential participants refused participation. Second, the current data information reached data saturation. In other words, once the researcher reached or interviewed a certain number(s) of participants, no new data information was merged. In this case, once the researcher reached 54 participants, no new data information was collected. However, in order to confirm data saturation, four more interviewees were invited. However, no new data information was collected. As a result, 60 participants were invited.

As this study covered most of the regions and cities in South Korea, the face-to-face interview sessions were not easy to be conducted. Also, the working schedules of the participants could be changed due to the ad hoc arrangement. However, due to the development of the technological and internet-based social media application, the researcher might employ social media chat interview technology for the interview session and data collection procedure. In this case, the researcher employed the Kakao Chat Application as the tool for the virtual-based interview sessions.

Due to concerns of personal privacy, the researcher needed to provide a pseudonym to each participant in order to protect their personal identity to other potential employers, current supervisors, government agencies, and even patients [55,56]. As the name of the employers (i.e., name of the hospitals or clinics), place of origin, and specialization would not impact the results of the findings and discussions, the researcher decided to mask irrelevant information. Please refer to Appendix A for the demography of the participants.

### 2.3. Data Collection

All of the participants had the right to withdraw from the study at any time without any punishments as the participation was totally voluntary. However, all decided to join the study without any withdrawal.

The qualitative data collection tools have been employed to collect data from the participants, including the semi-structured interview questions and the open-ended questions in the interview sessions [57,58,59]. During the interview session(s), the participants of this study were always encouraged to share their understanding, experiences, self-efficacy, and sources and reasons for stress and burnout. Under the guideline of the phenomenological approach [52], the researcher employed the general inductive approach as the tool to collect data [60]. Appendix B listed the interview questions. The interview questions were developed based on the self-efficacy approach [6,7,39] and the objective of this study.

As the researcher would like to collect rich and in-depth data from participants, the researcher decided to invite each participant for three interview sessions. According to Seidman [61,62], a relationship should be built between the participants and the researcher in order to capture the information. Although the researcher did not know all of the participants due to the limited personal networks and connections (i.e., snowball sampling strategy), three sessions of the interview sessions increased the understanding and relationship between each other. The interview sessions lasted between 60–99 min each.

One of the concerns for qualitative research was validity. Therefore, to confirm the validity of the data, after the data collection and analysis procedures, the researcher sent the related transcripts to the relevant participants for confirmation as the member checking procedure. As a result, all participants agreed on their parts for further reporting.

All of the participants were native Korean language speakers. However, due to the nature of their positions, all can speak the supreme level of English language. Moreover, the participants might have requested an interpreter (i.e., the researcher was responsible for the fees). However, none of the participants requested the interpreter. As a result, the conversations were recorded in English.

### 2.4. Data Analysis

After the progress of data collection from each participant (i.e., interview sessions), the researcher collected more than 500 pages of transcripts based on the oral conversations. Qualitative researchers [1,55,56,63,64] believed and indicated that big data and written qualitative transcripts should be reduced into meaningful themes, subthemes, directions, and groups [52,53].

First, based on the direction of the theoretical framework (i.e., self-efficacy approach) [51] and the suggestion from Merriam [55], raw qualitative data should be reduced for further development. The researcher reread the transcripts and related materials multiple times in order to find out the connections in between. As many participants shared different ideas, understanding, and lived stories based on their organization, department, life experience, location, and background, the researcher followed the guidelines of open-coding strategy [56] in order to reduce the data information into the first-level themes and subthemes. For the first-level themes and subthemes, there were 44 first-level themes and 66 subthemes reported. In fact, the large numbers of themes and subthemes were based on the rich and colorful experiences of the participants. However, based on the recommendation of Thomas [60], the overall themes and subthemes should be limited to no more than ten themes. Therefore, the second step had to be conducted.

Second, it is important to reduce the numbers into reasonable groups and themes. Based on the direction and method of the axial-coding strategy, the researcher reduced the first-level groups (i.e., themes) into the second-level themes. In sum, the researcher grouped four themes and four subthemes as the finding. For detailed information, please refer to Table 1.

### 2.5. Human Subject Protection

All of the signed and unsigned agreements, sensitive contacts, oral recording, transcripts, computer, and related materials were locked in the password-protected cabinet. Only the researcher had access to read the materials. After the study was completed, the researcher immediately destroyed and deleted all related materials for personal privacy.

As per the content forms and agreements, the place of origin, name of the employers (i.e., name of the hospitals or clinics), age, year of experiences, and specialization were masked due to privacy. Due to the small population and closed professional network, the researcher needed to protect the information of the participants. All subjects gave informed consent for inclusion before they participated in the study. The study was conducted in accordance with the Declaration of Helsinki, and the protocol was approved by the University Ethics Committee (2019/0203) and (2019/03332). The research was supported by Woosong University.

## 3. Results and Discussions

Although all the participants worked in a similar clinical environment (i.e., clinics and hospitals) in South Korea, their lived stories, experiences, self-efficacy, and sources and reasons of stress and burnout were not the same [1,65]. Therefore, it is important to categorize the participants’ answers in order to share their ideas with international readers.

Unlike previous studies [1,65,66,67,68,69,70] with a general perspective, this study focused on how gender and the career pathway as nursing professionals influenced the experiences, sources, and reasons of stress and burnout. The findings were categorized into four themes and four subthemes. Although the researcher expected the focus to be on overtime working hours and overload of responsibilities, most focused on issues regarding workplace bullying, family stress, and being misunderstood by members of the public. The results showed that all participants believed they would experience an overload of responsibilities due to the nature of their position (i.e., as nursing professionals). Therefore, most found that the negative experiences, sources, and reasons for stress and burnout came from the environment beyond their nursing responsibilities. Table 1 outlines the themes and subthemes of this study.

The researcher tended to capture how stress, burnout, overloaded responsibilities, social bias, and stigma influenced and impacted the working environment and how it influenced the self-efficacy, stress, and burnout related to nursing professionals’ working responsibilities in South Korea. Based on the data, sharing about the overload of working responsibilities was not significant. However, nearly all related sharing focused on discrimination and blaming from the people around them, even family members [1].

### 3.1. Workplace Bullying

#### 3.1.1. Discrimination from Doctors, Therapists, and Organizational Leaders

By understanding the sharing and experiences from the individuals, the researcher grouped several significant elements that impacted their experiences, particularly their self-efficacy, and sources of stress and burnout [1,6,7,39,40,41,42,43,44,51,65,66,71]. The researcher discovered that all participants indicated that the quality of their workplace environment decreased significantly due to their unbalanced working hours and overloaded responsibilities, for example, the mandatory overtime hours. Using the self-efficacy approach, the researcher discovered that all participants had experienced workplace bullying from different coworkers and employers. Several, who experienced mandatory overtime working hours without their approval, said,

*I have a family and I have many family responsibilities…. I have to take care of my family and my children…. I have two children; they are still in kindergarten…. I can work overtime, but I have to tell my mother in advance to take care them. However, my employer forced me to stay at the office without asking me…. I needed to go…, but if I left, I would have lost my job in three months… after working my notice….* (P#4)

*I do not want to work overtime as I have other responsibilities after working hours…, but my supervisor did not ask my approval or ideas…. I just need to cover the next eight hours without any agreements…. This is not a single case…. It always happens in Korea…. We have no rights as nurses….* (P#47)

*It is so unfair because the upper leadership always asks us to contribute and spend time with our patients…, but we are not machines…. I have parents and family members…. I still need to spend my time and my energy with my family…. I cannot always work overtime without any rewards….* (P#50)

All other participants shared similar experiences about ad hoc working responsibilities and an overload of working hours without their approval. All said that they understood the current extreme situation, but discussion and communication were greatly needed before any decisions were made. One participant, who felt any arrangements without approval was a disgrace, said,

*I don’t understand how come management cannot ask us first…. Of course, we can work overtime in this situation…, but I need to call my mother to ask her to take care of my children…. She needs to go over to my house to take care of my children…. This is a basic responsibility…, but management just forced us to work 24/7 in the office…. I always call my mother. At least management should call me…, allow me to make arrangements….* (P#6)

In this study, the results indicated that the irresponsibility, discrimination, and blaming from others became major sources and reasons of stress and burnout for mid- and senior-level nursing professionals in South Korea [64,72,73]. All 60 participants expressed that discrimination from other professional coworkers was one of the biggest problems in terms of low levels of self-efficacy, stress, and burnout [39,40,71]. Although the researcher asked about how such working conditions and overload in medical-related responsibilities would influence their mental and physical conditions, none of the participants expressed any negative experiences in this regard. In other words, they all accepted the overload in medical responsibilities as they are nursing professionals [72].

When briefly sharing about the satisfaction they got from their work responsibilities, many expressed a high level of self-efficacy and positive outlook. However, most could not accept discrimination from coworkers [27]. First, many indicated that the doctors they were partnered with always took advantage of them (i.e., nursing professionals), giving them non-related responsibilities, such as providing a personal massage after work, as two nursing professionals said,

*It would have been fine if the doctor had asked me to go to the ward to see to reports and patients…, but the doctor I am partnered with asked me to give him a massage after work…. I think this is totally beyond my responsibilities…. He didn’t do it politely; he forced me to do it…. I refused…. Then, I was transferred, partnered with another doctor….* (P#11)

*My partnered doctor always asks me to make him coffee in the morning and afternoon…, but I have patients and my break time is only less than 60 min…. I have to eat and rest…, but it seems like many doctors and male management love to take advantages from female nurses and other female staff….* (P#47)

Such excessive requests from doctors were not isolated cases in the South Korean medical environment. Another nursing professional said,

*In this extreme situation, my responsibility is to take care of the patients…. The workload was still extremely high due to the panic in the community…, but the doctor I am partnered with asked me to wash his shoes and uniform as he dropped coffee on himself…. It was an immediate request…. Should I take care of my patients or the doctor?* (P#15)

Besides doctors making excessive requests of participants, during the interview sessions, many expressed negative lived stories about the therapists they worked with. It was surprising that so much discrimination from coworkers was prevalent in clinics and hospital environments. For example, many expressed that some speech and audio therapists did not want to examine the throats and mouths of their patients and asked the participants to do it for them. As two said,

*Everyone wants to have a high level of protection. We all knew the nasal and oral openings and secretions could be very dangerous…, but we, as medical professionals, should take care of our patients under the guidelines from the department…. If need be, we should transfer the patients to the designated hospital…, but to keep asking others to do the dirty jobs they don’t want to do, such as cleaning nasal secretions on the floor…, this is totally beyond my understanding….* (P#25)

*Taking care of our patients is one of the most important jobs as public health professionals…. However, some staff always take advantages of nursing professionals as they don’t want to take any risks…, but if you don’t want to take risks…, why would you ask other people take these risks for you? I have family too. Is it because my life is cheaper as a female nursing professional?* (P#41)

With the reflection of previous studies [21,25,26], Korean organizational leaders and employers liked to abused their employees due to their leadership styles, especially medical professionals with a lot of excuses. Many were forced to work overtime without additional salary and compensation. Furthermore, the management did not ask for any approval from the medical professionals before making such arrangements. For example, more than two-thirds said that their management always used a sense of nationalism (i.e., being a patriot) to force them to do voluntary medical work. One participant said,

*Doing voluntary work is meaningful…. I love it…, but I should only do it if I want to…. I still have other personal arrangements after work…. I can do whatever I want after work…. It is my right to decline doing voluntary work…. It is not in my contract…, but my director forced me to do so without asking me at all…. He told me that this is a Korean practice….* (P#33)

Based on the findings, many said that the doctors and therapists they were partnered with and the organizational leaders always forced them to do additional work, particularly low-responsibility work, without their approval. Based on the self-efficacy approach [39,40,71], these sources and reasons of stress and burnout always increase their anger and confusion but reduce their self-efficacy as they still face such problems in their workplace every day [39,40,71].

#### 3.1.2. Discrimination from Other Nursing Professionals

Besides general hospitals, nursing professionals also work in different environments and organizations, infectious disease hospitals, general hospitals, clinics, cared housing facilities, community centers, and even school environments. However, social-levelling discrimination from nursing professionals at different facilities also caused them stress and burnout due to the unbalanced working responsibilities [39,40,71].

For example, several community nurses (i.e., in the clinics) were discriminated against by their nursing classmates and peers due to their safer working environment. Community nurses spoke about the bullying and blaming from their peers; for example, one said,

*Although I did not need to work with children…, community members…, it did not mean I was free…. I still had to do a lot of health promotion and community health activities for the public…. We all work in different positions in the city…. I don’t understand why people think we have it easier….* (P#31)

*For the coming three years, I am taking duties as the health promotional nurse at the community level…. Usually, I go to the community centers and schools for health promotion…; however, some of my coworkers always look down me because they think my duties are easier than theirs…. It is not easier to work with community members…. I don’t understand why people love to discriminate people based on their units….* (P#43)

Participants working in private clinics were also blamed by their peers due to their responsibilities and working environment; one participant said,

*I don’t know why peers in the hospitals like to blame us or discriminate against us…. We are working for the community…. We should not pigeonhole people…, but in Korea…, people like to pigeonhole others…. I was discriminated against…, but I will not discriminate against other people and other nursing professionals….* (P#34)

Based on sharing, it is worth noting that Korean people like to pigeonhole others based on their working environment, responsibilities, specialization, salary, working hours, and years of experience [39,40,71]. Although all participants said that they will not discriminate and blame people, the researcher captured a lot of discriminate sentences from the interview sessions. As a result, such discrimination and blaming always cause stress and burnout.

### 3.2. Family Stress

#### 3.2.1. Discrimination and Blaming from Family Members

According to the East Asian tradition regarding family arrangement, different generations should live together as a family unit [74]. In other words, besides the traditional parent–children family unit living together, tradition encourages eldest sons to practice the ideals of filial piety by taking in his parents as part of the household [75,76,77]. In this case, all 60 participants lived with at least one parent-in-law in their family. Many previous studies [17,78] indicated that the conflicts caused by the relationship with in-laws always increased tension and the number of arguments in the family. In this study, based on the sharing from the participants, the researcher categorized three types of arguments, discrimination, and blaming from family members, which were conflicts with in-laws, blaming from husbands, and ignorance from children [39,40,71]. It is worth noting that the conflicts among family members were upgraded. The following sections and parts explained and explored this issue.

In this case, all 60 participants expressed different opinions about how family members discriminate against them based on their occupation as nursing professionals who need to interact with different types of patients in the South Korean medical facilities [79]. Seen through the lens of the self-efficacy approach, many have low levels of self-efficacy due to both mental and physical discrimination from their family members, which are highly related to their sources and reasons of stress and burnout. In other words, even after the participants left their high-pressure working environment after working their hours, the mental pressure was not relaxed as their family members continued to look down on them. For example, several participants shared the following:

*Even if I finished work at the hospital…, when I returned home, my mother-in-law, my husband, and my sons asked me to eat alone in the kitchen and asked me to sleep in the storage room as I am a dirty person…. Society called us heroes, but this is not true…. Society always discriminated against us because of our occupation, our contact with patients….* (P#28)

*My mother-in-law and my husband always look down me because I need to work in the dangerous unit…. When I enter my house, my mother-in-law asks me to wash my body before touching anything in the home…. My husband even asks me to isolate myself before I go to the bedroom….* (P#44)

First, all 60 participants experienced extreme cases, discrimination, and verbal harassment from their in-laws. Many said that their mother-in-law always called them dirty women because they worked at the hospital, particularly some participants who work in infectious disease units. One such participant said,

*My mother-in-law called me a dirty woman because I was sent to the infectious disease patients testing unit…. After I leave the unit, I even… clean up my body… in order to ensure my safety and the safety of my family…, but when chatting with my family online, I heard my mother-in-law calling me a dirty woman…. She used this term to describe my occupation in front of my sons….* (P#23)

Another participant also shared a similar experience, saying,

*It is all about my occupation as a nursing professional…. I feel I am working for the good of my region…. Although I don’t make additional money or whatever, I serve the patients in need and provide help to those who need me…, but my mother-in-law does not understand…. She told me to go into rural housing for at least a month before returning….* (P#20)

These were not isolated cases, with multiple cases being reported by all 60 participants. A misunderstanding from family members always causes a low level of self-efficacy, stress, and burnout, as they do not receive approval from the loved ones in their family. This disapproval, seen through the lens of the self-efficacy approach, always causes stress and burnout [39,40,71].

Second, based on the traditional East Asian perspective, married women should always stay home to work as full-time housewives [25,26,80,81]. However, due to financial pressures, many women need to work outside the home too. All of the participants are in this double-working situation. They indicated that, due to financial pressure (e.g., husband cannot earn enough money for the family), they need to work in medical and nursing professions after marriage.

When children and school-age students did not need to go back to school, many women nursing professionals faced conflicts and misunderstanding from their family members. First, several commented about their extraordinary support for their family after marriage; one said,

*I have worked ever since my marriage in order to compensate for my husband’s low earnings…. I do not complain as I want to support my family…. Even if he doesn’t support the fact that I work after our marriage, I think working for patients as a nurse is very meaningful…, but now, he thinks I am a dirty woman because I have to touch patients…. Once, he asked me not to come back home….* (P#10)

This sharing indicated one of the strongest sources of low self-efficacy, stress, and burnout as her husband strongly discouraged her hard-working responsibilities for both family and the public health system [39,40,71]. Although the participant explained to her husband about her contribution to the government and the region, he continued to call her a dirty woman. Many participants shared similar feedback; one said:

*My husband called me a stupid girl as I want to spend my energy working in the public health system…. He said that we should be selfish as we have already paid enough tax to the government…. The government should have the ability to manage the manpower…. He continues to call me stupid girl…, but even if the government has money, how can they have enough manpower if we don’t work…. Even if the government never ever respects the public health system workers, I still want to work for my family….* (P#7)

Third, not only were the relationships with their in-laws and their husbands affected but also many indicated that their children ignored their occupation and rejected the parent–child relationship in front of their classmates and friends. More than two-thirds of participants shared that their children looked down on them because of their responsibilities in the medical and nursing profession (i.e., caring for patients). More importantly, their children would rather say their mother was a housewife than a nursing professional in the medical facilities. Their children’s ignorance served as a source of low levels of self-efficacy, stress, and burnout related to their occupation [39,40,71]. For example, two said:

*My son did not talk to me because he thinks I am a dirty worker in the hospital…. I told my husband to tell him that my work is exclusively for the public health system and patients…, but my husband did not listen to me and said a lot of bad things to my son…. My parents…, the same…, they decided to keep their silence….* (P#22)

*My children did not call me mother because I am a nursing professional in a hospital…. My mother-in-law and my husband always tell my children that I work in the dirty unit in the hospital…. I do not think working in a dirty unit is an ugly thing as a public health professional…. I am professional…. I work for people who are sick….* (P#26)

Seen through the lens of the self-efficacy approach, under this theme, it is worth noting that the participants’ low self-efficacy was mainly caused by misunderstanding, disapproval, and ignorance from their family members. Although many spent their energies to contribute to the public health system, no one in their family understood and admired their hard work. These negative reactions from their family members caused low self-efficacy.

### 3.3. Being Misunderstood by Members of the Public

Besides the pressures from the workplace and family members, the general public and communities also disagreed on the hard work they were doing for the public health system [1,65,81,82]. Based on many previous studies, it may be said that Koreans always believed in blaming others and pigeonholing people based on their characteristics, such as occupation, social status, and sexual preference. Therefore, based on these Korean traditions and practices, besides coworkers and family members, it is not uncommon that members of the public discriminate against and misunderstand the nursing profession [39,40,71].

#### 3.3.1. Blaming from the Public

First, at the regional level, many indicated that, in the newspapers and on news channels, many local residents blamed medical and nursing professionals for not spending enough energy to protect civilians. Several indicated that news channels always interviewed people who disliked women who work [80,81], especially married women; two participants said,

*Many newspapers like to catch their readers’ attention…, always printing negative and fake information about the public health system…. Korean people like to blame others in order to diminish their responsibilities… even in this public health disaster….* (P#1)

*It is not uncommon for gender-based discrimination in Korea…, but I feel so negative because society discriminates us based on our gender and occupation…. I do not think nursing professionals should be discriminated…. We work so hard to help the patients…, but the rewards from the community are negative.* (P#38)

Seen through the lens of the self-efficacy approach [39,40,71], many believed that the blaming tradition in Korean society reduces their self-esteem and self-efficacy as nursing professionals and increases their stress and burnout levels.

Second, at the community level, many participants indicated that, in their neighborhood, many people and neighbors told and transferred gossips about participants’ occupation (i.e., nursing professionals) for others in order to avoid and discriminate interactions and connections with the participants and their family members. In other words, many community members labelled nursing professionals and their family members as aliens, out of ignorance [33]. One participant said,

*In our building, we have a social media chat group…; however, my family members… and my neighbors… were forced to leave the chat room… as Koreans like to pigeonhole people… in this case, based on this public health situation….* (P#3)

Another participant said that she understood this isolation as she had been one of those who discriminated against and socially stigmatized other minorities in her neighborhood,

*I understood why my neighbors forced us to leave their chat room as we used to force other disabled, senior citizens, and families with special needs children to leave us…. This is the Korean way…. I do not wish to change this behavior as I think it is not essential. I will discriminate against others in return in the future in order to reassert my social status….* (P#17)

Although many participants indicated that they are fighting for social justice and equality in their community, many were discriminatory and biased themselves due to their social status and background (i.e., religious practices and occupation). Therefore, within Korean society, discrimination towards others is not uncommon due to the customary pigeonholing and grouping. In this case, many advocated that their self-efficacy, stress, and burnout were the result of interaction with the community.

### 3.4. Personal Development and Career Enhancement

Although many participants expressed negative feeling and lived stories about their career role and responsibilities as nursing professionals in South Korea, a few positive points were captured. In this theme, the researcher grouped that many participants were encouraged to study some professional development courses and postgraduate degree programs in order to promote the image of clinics and hospitals. Although the organizations always encourage their staff and professionals joining the study programs, different feeling and perspectives were shared. First, a group of participants believed that the results of their studies may increase their chances for promotion, one said,

*If I graduated with a master’s degree nursing, I may be promoted as one of the team leaders…. My salary will be increased…. Also, I can escape from my current hospital… and look for a better working environment…. At least I have the chance to change my job….* (P#47)

These participants advocated that their education may be connected to their salary and career development. However, another group of participants expressed other concerns about the educational achievements of these study opportunities. With the reflection of a previous study [21], the social level and organizational hierarchy are not uncommon in the South Korean working environment. Many expressed that, in South Korea, nursing professionals are viewed as the affiliated staff of the doctors and physicians. Therefore, if the nursing professionals received higher qualifications and skills than the doctors and physicians, concerns of social level and organizational hierarch could happen, as one said,

*As I explained above, workplace bullying is not uncommon in South Korea…. I wish I can study a master’s degree or a doctoral degree… and become a better educated person…, but if my supervisor and organizational leaders knew I have a postgraduate qualification…. I am not sure about their reactions…. I fear actually….* (P#50)

In conclusion, based on the self-efficacy approach [39,40,71], the low level of self-efficacy among nursing professionals mainly comes from discrimination and social bias from their coworkers, family members, and members of their communities. A previous study [79] indicated that it is a traditional practice for Koreans to pigeonhole people based on their occupation, gender, family status, place of origin, and so on. Therefore, the results showed that low levels of self-efficacy and discrimination were interconnected. Although medical and nursing professionals contribute their energies and efforts to the public health system, their contribution did not change the perspective of the Korean people, thus continuing to reduce their self-efficacy [39,40,71].

## 4. Limitations and Potential Developments

This research study has four limitations. First, it could have covered additional public health and social caring professionals and workers to gain a better understanding. For example, future research studies may collect data information from doctors, therapists, health and social care educators, preservice health, and social care practitioners, and even supporting staff within the public health system. Without an overall and holistic picture, certain points and elements of the public health system could be missed.

Second, the present research merely covered data from 60 nursing professionals. Due to the population (i.e., limited personnel), the researcher could only recruit a small group of nursing professionals. Future research studies should interview additional participants and people with various backgrounds in order to increase the detail and content of the study.

Third, discrimination, social stigma, and social bias are ongoing problems in many countries and regions. Therefore, researchers may continue to research and compare these issues and problems in future.

Fourth, the current study collected data from individuals all over South Korea. However, future research studies may focus on a single province or city, such as Seoul, Busan, and Jeju Province, in order to conduct an in-depth exploration of the province or city. For example, individuals from rural communities may generate different data from metropolitan individuals. Therefore, future studies and research are needed.

## 5. Implications

Nursing professionals and related medical staff are important cogs in the public health system workforce. Although the nature of the positions cannot be changed immediately, the results of this study provided some directions for improvement. First, policymakers should review the current policy and regulations about the job specification of nursing professionals and related medical staff in order to provide appropriate guidelines for job responsibilities.

Second, external parties or professional associations should be established in order to investigate inappropriate behavior, mismanagement, malpractice, and bad leadership beyond traditional medical misconduct. For example, unions or associations for medical staff could be useful to protect the rights of employees.

Third, from the educational perspective, it is important to provide appropriate education for both youths and adults for them to understand the responsibilities related to different occupations and career paths. Although social levels, stigma, and discrimination exist in all communities, societies, parents, teachers, and individuals should understand the respectfulness owed to different individuals and groups in societies.

## 6. Conclusions

To the best of the current knowledge, this is one of the first self-efficacy, stress, and burnout studies that is based on the sharing and lived experiences of nursing professionals in South Korea. Based on sharing and feedback, most of the participants advocated that workplace bullying, family stress, and misunderstanding by members of the public were the major sources of reduction in their self-efficacy due to the stress and burnout elements. Based on the self-efficacy approach, although self-efficacy levels could be highly influenced by (1) personal factors and elements, such as cognition, affect, and biological elements; (2) behaviors; and (3) environmental elements and factors, most nursing professionals involved in this study indicated that environmental elements and factors (i.e., stress and burnout from their working environment) were the sources of stress and burnout. Therefore, the results of this study captured the overall working environment in South Korea and how nursing professionals described their experiences. Based on the self-efficacy approach, the environmental elements and factors related to their working environment were deemed to be important factors related to their self-efficacy issues.

## Figures and Tables

**Table 1:** Themes and subthemes.

Themes and Subthemes		
Section 3.1		Workplace Bullying
	Section 3.1.1	Discrimination from Doctors, Therapists, and Organizational Leaders
	Section 3.1.2	Discrimination from Other Nursing Professionals
Section 3.2		Family Stress
	Section 3.2.1	Discrimination and Blaming from Family Members
Section 3.3		Being Misunderstood by Members of the Public
	Section 3.3.1	Blaming from the Public
Section 3.4		Personal Development and Career Enhancement

## Appendix A

The demography of the participants

**Name**	**Location of the Workplace**	**The Setting of the Workplace**
P#1	Seoul	Hospitals
P#2		
P#3		
P#4		
P#5		
P#6		
P#7		
P#8		
P#9		
P#10		
P#11		Clinics
P#12		
P#13		
P#14		
P#15		
P#16		
P#17		
P#18	Incheon	Hospitals
P#19		
P#20		
P#21		
P#22		
P#23		
P#24	Daegu	Hospitals
P#25		
P#26		
P#27		
P#28		
P#29		
P#30		
P#31		
P#32		Clinics
P#33		
P#34		
P#35		
P#36		
P#37		
P#38	Daejeon	Hospital
P#39		
P#40		
P#41		
P#42		
P#43		
P#44	Busan	Hospitals
P#45		
P#46		
P#47		
P#48		Clinics
P#49	Gyeonggi-do	Hospitals
P#50		
P#51		
P#52	Gyeongsan-do	Hospitals
P#53		
P#54		
P#55	Gangwon-do	Hospitals
P#56		
P#57	Joella-do	Hospitals
P#58		
P#59	Jeju-do	Hospitals
P#60		

## Appendix B

The Initial Interview Questions

(1) Do you enjoy being a nurse? Why or why not?
(2) Is it hard to work as a nurse now? Why or why not?
(3) What would be the hardest thing or things you face as a nurse?
(4) What about now? What would be the hardest thing or things you face as a nurse?
(5) Further questions.

The Second Interview Questions

(1) Do you feel stress and burnout? What situations made you feel stress and burnout?
(2) How would you describe your current self-efficacy? Can you tell me more?
(3) Do any personal factors and elements, such as cognition, affect, and biological elements make you stressed and burnt out?
(4) Do any behavioral actions and ideas make you stressed and burnt out?
(5) Do any environmental elements and factors, such as working condition and issue, make you stressed and burnt out?
(6) Further questions.

The Third Interview Questions

(1) How would you describe being a nurse in South Korea based on your gender?
(2) How would you describe bring a nurse in South Korea based on your age?
(3) If possible, do you want to continue to work as a nurse? Why or why not?
(4) How would you describe the future development of the nursing profession in South Korea? For example, any improvements.
(5) Further questions.
